# Modeling personal particle-bound polycyclic aromatic hydrocarbon (pb-pah) exposure in human subjects in Southern California

**DOI:** 10.1186/1476-069X-11-47

**Published:** 2012-07-11

**Authors:** Jun Wu, Thomas Tjoa, Lianfa Li, Guillermo Jaimes, Ralph J Delfino

**Affiliations:** 1 Program in Public Health, College of Health Sciences, University of California, Irvine, USA; 2 Department of Epidemiology, School of Medicine, University of California, Irvine, USA; 3 Department of Environmental Science, Policy, & Management, University of California, Berkeley, USA

**Keywords:** Particle-bound polycyclic aromatic hydrocarbon, Personal exposure, GPS, Time activity, In-vehicle travel, Traffic

## Abstract

**Background:**

Exposure to polycyclic aromatic hydrocarbon (PAH) has been linked to various adverse health outcomes. Personal PAH exposures are usually measured by personal monitoring or biomarkers, which are costly and impractical for a large population. Modeling is a cost-effective alternative to characterize personal PAH exposure although challenges exist because the PAH exposure can be highly variable between locations and individuals in non-occupational settings. In this study we developed models to estimate personal inhalation exposures to particle-bound PAH (PB-PAH) using data from global positioning system (GPS) time-activity tracking data, traffic activity, and questionnaire information.

**Methods:**

We conducted real-time (1-min interval) personal PB-PAH exposure sampling coupled with GPS tracking in 28 non-smoking women for one to three sessions and one to nine days each session from August 2009 to November 2010 in Los Angeles and Orange Counties, California. Each subject filled out a baseline questionnaire and environmental and behavior questionnaires on their typical activities in the previous three months. A validated model was used to classify major time-activity patterns (indoor, in-vehicle, and other) based on the raw GPS data. Multiple-linear regression and mixed effect models were developed to estimate averaged daily and subject-level PB-PAH exposures. The covariates we examined included day of week and time of day, GPS-based time-activity and GPS speed, traffic- and roadway-related parameters, meteorological variables (i.e. temperature, wind speed, relative humidity), and socio-demographic variables and occupational exposures from the questionnaire.

**Results:**

We measured personal PB-PAH exposures for 180 days with more than 6 h of valid data on each day. The adjusted R^2^ of the model was 0.58 for personal daily exposures, 0.61 for subject-level personal exposures, and 0.75 for subject-level micro-environmental exposures. The amount of time in vehicle (averaging 4.5% of total sampling time) explained 48% of the variance in daily personal PB-PAH exposure and 39% of the variance in subject-level exposure. The other major predictors of PB-PAH exposures included length-weighted traffic count, work-related exposures, and percent of weekday time.

**Conclusion:**

We successfully developed regression models to estimate PB-PAH exposures based on GPS-tracking data, traffic data, and simple questionnaire information. Time in vehicle was the most important determinant of personal PB-PAH exposure in this population. We demonstrated the importance of coupling real-time exposure measures with GPS time-activity tracking in personal air pollution exposure assessment.

## Introduction

Airborne polycyclic aromatic hydrocarbons (PAH) are produced from incomplete combustion of fossil fuels and other organic materials [1]. Major ambient sources include heavy traffic and industry that use coal burning and smelting, while major indoor sources include indoor smoking, use of unvented heating appliances such as wood burning fireplaces and coal burning stoves, and some types of cooking [2,3]. PAH exposures have been associated with increased risks of systemic inflammation [4], cardiopulmonary mortality [1,5], lung cancer mortality [6,7], and adverse pregnancy outcomes (e.g. low birth weight, intrauterine growth retardation, and *in-utero* fetal death) [8-12].

PAHs are semi-volatile organic compounds and present in both gaseous and particulate phases in the atmosphere, depending on the vapor pressure of each PAH compound. Gas-phase PAHs (mostly low-molecular-weight PAH) may account for the majority of the total PAH mass in the urban atmosphere [13-15], but are considered less mutagenic and/or carcinogenic than high-molecular-weight PAHs that are concentrated in the fine and ultrafine particles [16]. However, a recent Europe study showed that both gas-phase and particle-phase PAHs (PB-PAH) may contribute significantly to lifetime lung cancer risk [17].

Inhalation exposure to PAH can be highly variable between locations and individuals [2]. In epidemiological studies, personal PAH exposures are usually measured through personal monitoring or biomarkers [1] although personal monitoring or biospecimen collection and analysis is costly and impractical for a large number of subjects. Centrally located monitors have also been used to estimate personal PAH exposure although this approach may underestimate exposure and between-person variability [18]. Another approach is to model PAH exposure based on limited personal or microenvironmental measurements coupled with personal time activity data [19], which offers a cost-effective means of estimating personal exposures without the logistical difficulties of personal or biomarker sampling.

The modeling of personal exposure to PAH has primarily focused on occupational exposures that tend to come from a single dominant PAH source and at work places only [20]. Challenges exist in modeling personal exposure in non-occupational settings because the PAH exposures may occur at different microenvironments (e.g. outdoor, indoor, and in-vehicle) and from multiple sources (e.g. traffic, industry, smoking, and cooking). Aquilina *et al.* (2010) developed and compared different models to estimate non-occupational personal exposure to PB-PAH. However, their study relied heavily on detailed questionnaire data on activities such as solvent use, particle generation, smoking (e.g. the number of smokers, number of cigarettes smoked, indoors or outdoors, and at which distance from the sampler these were smoked), ventilation modes at home, possible PAH sources in the garage, as well as indoor/outdoor locations and distance to the road. Although such detailed information is helpful in exposure estimates, it creates tremendous subject burden in epidemiological studies. Furthermore, similar to most of the previous studies on personal exposure modeling, the subjects’ time-location patterns were recorded on diaries [19], which may be limited by accuracy of recall, reliability, and compliance [21]. Recently, new techniques have been used to collect time-location data, such as the use of portable global positioning system (GPS) devices to track people’s time-location [21,22]. However, GPS tracking has seldom been used in personal exposure modeling, likely due to the difficulty of analyzing the large amount of GPS data [23].

For personal PB-PAH measurements, most of the previous studies have collected PB-PAHs on pre-baked filters [19,24,25]. This sampling approach has the advantage of measuring the integrated mass of different PB-PAH species, but it does not quantify exposures at a high spatial and temporal resolution. Some studies have measured real-time PB-PAH with a diameter below 1 μm in specific micro-environments (e.g. ambient outdoor, indoor, and in-vehicle) using the photoelectric aerosol sensor (PAS, EcoChem Analytics, League City, TX) [26-29]. The PAS employs photo-ionization by mean of ultraviolet light; positively charged particles are collected on a filter, generating a current that is measured by an electrometer [30]. Field studies have shown that there is a nearly linear relationship between the PAS signal and PAH levels measured in samples of particles filtered from ambient air [26], [31-33]. The PAS has the advantages of ease of data collection, real-time sampling, and relatively low costs compared to the methods that rely on the chemical analysis of airborne particulate matter. However, the PAS sampler only measures total PB-PAH and not individual PB-PAH species (e.g. benzo[a]pyrene and other carcinogenic compounds). Particles containing the same total mass of PAH may produce different photoemissions signals since the contribution of individual PAH to the total photoemission signal may not be directly proportional to their concentration on the particles [34]. For example, particles coated with benzo[a]pyrene produce strong photoemissions signals, while particles coated with chrysene are not easily photoionized [34]. Furthermore, although the PAS has been developed for PB-PAH measurements, the PAS signals can also be correlated to other traffic-related compounds like soot and elemental carbon [35] since the PAS works on the principle of photoionization rather than the chromatography-mass spectrometry procedures. Because of the potential limitation above, the PAS was described as a semi-quantitative tool for PB-PAH estimation [36]. However, no better instruments were available that can measure real-time personal PB-PAH exposures in human subjects. Finally, most of the previous studies with real-time PB-PAH measurements focused on micro-environmental exposure rather than personal exposure. This is likely due to the limited battery life (4–6 h) of the portable PAS device [37]. The short battery life can be circumvented by replacing the original battery with a longer lasting battery.

The main aim of this study was to advance our understanding of the causes and magnitude of exposures to PB-PAH by major time-activity patterns of human subjects and develop models to estimate personal PB-PAH exposures through information that can be easily collected using GPS tracking. While there are several other sources of PB-PAH in southern California, we focused on personal PB-PAH exposures due to traffic-related sources among human subjects. No gas-phase PAH was examined in this study. The target of the present study, Los Angeles Metropolitan area in Southern California, has been one of the most polluted places in the country [38]. The urban core of the area (Los Angeles-Long Beach-Santa Ana) had a population density of 2,702 inhabitants per km^2^[39], encompasses the nation’s largest marine port complex [40], and has six major commuter and truck transport freeways. The 15 million registered on-road vehicles in the greater Los Angeles Basin are among the largest contributors to fresh emissions of PB-PAHs [13].

## Methods

### Subjects recruitment

This study was embedded in an air pollution and pregnancy outcome study funded by the National Institute of Environmental Health Sciences. We recruited 92 pregnant women in Los Angeles or Orange Counties of southern California in 2009–2010. The subjects were enrolled before 20 weeks of gestation at two hospitals (Long Beach Memorial Medical Center and the University of California, Irvine Medical Center). Eligible subjects were 18 years of age or older, nonsmokers and experiencing low-risk pregnancies (e.g. excluding those with illegal drug use, alcohol abuse, hypertension or diabetes before pregnancy). The study protocol and material were approved by the University of California, Irvine Institutional Review Board for biomedical research.

### Sampling methods

Among the 92 subjects, 28 women participated in the personal PB-PAH exposure sampling coupled with GPS time-activity tracking for one to three times and one to nine days each time from August 2009 to November 2010. The sampling was performed either during the pregnancy or after the women delivered the baby. The subjects carried a compact (3-lbs) PAS 2000CE sampler (EcoChem Analytics, League City, TX) in a backpack with a GlobalSat DG-100 GPS device (approximately 227 g) during waking hours. The PAS samplers were set to sample every 20 s and output the average concentration of three readings in one minute. The battery life of the PAS was extended from the original 4–6 h to approximately 16 h by re-engineering the battery of the device by the EME Systems (Berkeley, CA). The participants were instructed to leave the device plugged in while at home to charge the PAS and the GPS device. The charging cord was set up by our research staff in a central location in the home away from obvious sources of smoke (e.g. away from the stove or areas where candles were lit). The PAS samplers were factory calibrated before use. The PAS had a detection limit of approximately 1 ng/m^3^ and a measurement range of 0–4000 ng/m^3^. The sensitivity of the PAS was around 1 ng/m^3^ for 1 femtoamp – the raw signal the PAS measures. The DG-100 has been demonstrated as a reliable GPS device in tracking time-locations of human subjects [41]. The PAS sampler was synchronized with the time of the GPS device. The PAS was set to sample every one minute, while the GPS device recorded every 15-s.

In addition to the personal exposure monitoring, each subject filled out a baseline questionnaire on demographic and socioeconomic information, including age, race/ethnicity, reproductive history, height and weight before pregnancy, education, annual household income, marital status, and primary language spoken at home. Furthermore, the subjects were administered a questionnaire right before each personal-exposure sampling session on the major environmental and behavior patterns that may influence their exposure to PB-PAH in the past three months of the interview day. This environmental and behavior questions collected information on home location, housing characteristics (e.g. house age, gas stove used in cooking, use of air conditioning, gas heaters), work locations, and transportation means from home to work and back. To reduce the burden to the subjects, we did not ask subjects to keep a log of the time spent in each microenvironment, opting instead for more accurate GPS methods.

### Time-location classification

The GPS latitude and longitude coordinates were transformed to North American Datum (NAD) 83 and Zone 11 N projection. We then classified the GPS points into three major time-activity categories: indoor, in-vehicle travel, and other using the automated method we describe elsewhere [23]. We examined the model performance using sensitivity [the ability of the model to identify specific cases: true positive estimation/(true positive estimation + false negative estimation)], specificity [the ability of the model to identify non-cases: true negative estimation/(true negative estimation + false positive estimation)], and precision [the proportion of predicted cases that are correctly real cases: true positive estimation/(true positive estimation + false positive estimation)]. With good-quality training and validation data, we reported that the model had 94.8% sensitivity, 82.6% specificity, and 98.2% precision in identifying indoor GPS points, and 87.8% sensitivity, 99.5% specificity, and 89.1% precision in identifying in-vehicle travel points, respectively [23]. We grouped the outdoor static and outdoor walking categories from the model output into the “other” category since we found substantial uncertainty in classifying these two activity categories. Next, we counted the number of each time-activity categories (based on the 15-s data) in one minute and assigned the category with the highest count to the 1-min time-activity classification. Accordingly, the coordinates, speed, and altitude were also averaged at a one minute interval. Finally we linked the GPS-based time-activity classification with the PB-PAH exposures by subject, and date and time of the measurements.

### Data quality assurance

First, we overlaid the GPS points with roadway data in ArcGIS (ESRI, Redlands, CA) and visually checked the model-estimated in-vehicle travel points. We obtained a total of 195,232 min of valid PB-PAH exposure data. A total of 2305 records from three subjects were removed since they were likely misclassified as in-vehicle travel (i.e. clustering of GPS points with no apparent pattern of in-vehicle travel); 99.1% of the removed data came from one subject who lived in a big gated apartment complex and our time-activity classification model may have misclassified outdoor walking to in-vehicle travel. We found five 1-min indoor concentrations above 1000 ng/m^3^ in three subjects, which were likely caused by measurement noise or exposure to indoor sources such as environmental tobacco smoking (ETS) or cooking (e.g. grill or barbecue). The five points were excluded from the analysis since we focused on PB-PAH exposures from traffic-related sources and did not administer detailed time-activity logs to identify indoor activities. Further, we examined the completeness of the data based on the number of 1-min PB-PAH measurements on each day. The incomplete days of data were mostly likely caused by the subject non-compliance (e.g. they forgot to charge the battery) and the battery failure of the PAS sampler (one subject-session) and the GPS device (three subject-sessions). Finally, we excluded 3.7% of the data (N = 7260 min) that lasted for less than 6 h on a particular day (range 1 to 357 min).

### Co-variables

We examined seven major groups of variables, including time-activity patterns (either the modeled value or using GPS-based speed as a proxy for in-vehicle travel), roadway and traffic covariates, meteorological parameters, day of week and time of day, subject’s vehicle information, demographic and socioeconomic variables, and occupational exposure. These variables were selected because they directly or indirectly reflected the strength of emissions, subject’s proximity to the sources, potential source reduction (e.g. newer vehicles likely have lower air exchange rates and reduced PB-PAH penetrated from outside), and the impact of meteorology on the air quality.

We obtained roadway data for the study region from the ESRI StreetMap™ North America 9.3 (http://www.esri.com). This dataset was bundled with ArcGIS software products and included 2003 TeleAtlas® street data rather than the less-accurate TIGER 2000-based street data [42]. We obtained the 2002 annual average daily traffic (AADT) count data from the California Department of Transportation (Caltrans) with continuous coverage of total traffic counts on freeways, highways, and major arterial roads. AADT was produced by Caltrans staff based on a combination of measurements and modeled values as an alternative to limited traffic counts. Traffic density was calculated using the Kernel Density function of Spatial Analyst in ArcInfo GIS 9.1 (ESRI, Redlands, CA). Previous measurement studies indicated that ultrafine particles (UFP) and carbon monoxide dropped to near-background levels at 200 m downwind from major roadways during daytime hours (10 AM – 6 PM) [43] and up to 2000 m downwind during pre-sunrise hours (4:00 – 7:30 AM) [44]. There is no perfect cut point to define the size of a traffic influenced zone due to changes in roadway pollution dispersion depending on time of the day and atmospheric stability. In this study, traffic density was calculated at a 20x20 m resolution using a search radius of 300 m and 500 m. A smooth curved surface of traffic volume was fitted over each road segment using adapted quadratic kernel function; the value was greatest on the road, diminished as it moved away from the road, and reached zero at the search radius distance from the road (perpendicular distance). We also calculated length-weighted AADT (i.e. ∑(AADT*roadwaylenght)/Σ(roadwaylenght)) within 500 m of each sampling point and assigned the type of roadway (i.e. freeway and highways vs. other streets) and the distance to the nearest roadway to each point.

Ambient hourly wind speed, temperature, relative humidity, and precipitation were obtained from the nearest weather monitoring stations operated by the National Weather Service. Since little variability was observed in the precipitation data on the sampling days, the precipitation variable was excluded from further analysis. We classified day of week by weekday and weekend, time of day by daytime (6 AM – 7 PM) and night time (7 PM – 6 AM), and by rush hours (6–8 AM and 4–7 PM) and non-rush hours (all other times). We calculated the percentage of PB-PAH data that were collected during the weekday, daytime, rush hours, indoors, and while in-vehicles separately by person-day and by person. We also examined the impact of the subjects’ socioeconomic status (i.e. household income and language spoke at home), which may directly influence the subjects’ behavior patterns and subsequently their proximity to emission sources. Furthermore, we looked at housing characteristics, vehicle type and mileage, pregnancy status, working status, and occupational exposure to traffic-related pollutants. Due to the limited number of subjects who reported occupational exposure, we consolidated positive answers to work-related exposure by including working around a parking garage, kiosk, auto shop, buses, trucks, or heavy traffic, or driving a car/bus/truck during the work day.

### Data analysis and model development

We analyzed the data and developed models using SAS v9.2 (SAS Institute Inc., Cary, NC). We treated the following as continuous variables in the models: GPS speed and altitude, AADT on the nearest road, length-weighted AADT, traffic density, the percentage of weekday, daytime, time in rush hour, time indoors, time in-vehicles, and ambient meteorological parameters (temperature, wind speed, and relative humidity). Here the GPS speed was the speed recorded by the GPS device, which reflected the moving speed of a subject. The following parameters were treated as categorical variables in the models: day of week, age, household income, language spoken at home, rented vs. owned home, cooking stove (gas vs. electric), vehicle type (Asian vs. German vs. U.S. manufactures), mileage (>100,000 miles vs. ≤100,000 miles), roadway type (freeway and highway vs. others), pregnancy status (yes/no), working status (yes/no), and occupational exposure to traffic-related pollutant (yes/no).

Summary statistics were computed for minute-level data and data at different averaging periods (e.g. person-day and the subject’s entire sampling set). We decided to develop predictive models for each subject’s daily exposure and exposure across all of their sampling sessions (1–3 sessions and 1–9 days per session) since there were significant temporal and spatial autocorrelation in the minute-level data. More importantly, daily and longer-term exposures are expected to be more meaningful than minute-level data in most epidemiological studies focusing on health outcomes that are chronic (e.g. cancer), sub-chronic (pregnancy outcomes), or even acute (e.g. daily asthma morbidity). The PB-PAH exposures were time-averaged or time-weighted by three methods: 1) by subject and day of measurements; 2) by subject and time activity categories (i.e. indoor, in-vehicle, and other) across all the sampling sessions; and 3) by subject across all the entire sampling sessions. For each of the averaging methods, the continuous covariates were also averaged accordingly. For the categorical variables, we matched the session-specific data (e.g. rented vs. owned home, cooking stove, pregnant and working status, and occupational exposure) to PB-PAH exposures by subject and session of measurement. For averaged PB-PAH exposures across all the sampling sessions, we assigned the categorical variables collected at baseline of the first sampling session; sensitivity analysis showed that the inclusion of these variables collected at different sessions (if available) had little influence on the results.

We developed multiple linear regression models to predict average personal daily PB-PAH exposures and subject-level average PB-PAH exposure (total and by time-activity categories). We also fitted a linear mixed effect model with a random intercept and random slope for each subject to account for subject-specific variations in daily PB-PAH exposures. We applied the square root function to transform the daily and subject-level exposures to a normal distribution. Scatter plots were used to examine the linear or non-linear relationship between the square-root transformed PB-PAH exposure and the covariates. An apparent non-linear relationship was identified for GPS speed, thus the square root of speed was used in the model because it was more linearly correlated with PB-PAH (Additional file 1: Figure S1). For model development, we first examined the correlation of each variable with daily PB-PAH concentrations. A variable was dropped from further analysis if the absolute correlation coefficient with the measured concentrations was less than 0.10. Highly correlated variables (r ≥ 0.8) were examined separately in the model. We used the LASSO method of variable selection [45] in the SAS GLMSELECT procedure to select the best-fit model. The covariate combination with the maximum R^2^ or minimum Akaike’s information criterion (AIC) was selected as optimal inputs in the model. The models selected were further checked by the variance inflation factor statistics (VIF) to assess potential colinearity.

## Results

### Pollutant concentrations

We collected 180 person-days (N = 185,662 min) of real-time PB-PAH exposure data with valid GPS time-activity classification. The geometric mean of PB-PAH exposure per subject was 9.3 ng/m^3^ with a standard deviation of 1.9 ng/m^3^. The raw PB-PAH exposures were highly skewed to the right for minute-level exposure (skewness = 8.76), and somewhat skewed for daily exposure (skewness = 1.74) and subject-level exposure (skewness = 1.10) (Additional file 1: Table S1). The square root transformation gave a more normal distribution that the log transformation (Additional file 1: Table S1). Among the 28 subjects, 22 of them had more than one day of measurements (range 2 to 22 days) (Table 1). We observed significantly higher (p-value < 0.05) PB-PAH exposures under conditions of higher traffic density measurements and lower relative humidity when PB-PAH was compared between the upper and lower half of the distributions of the two variables (Table 1). Higher PB-PAH exposures of borderline statistical significance (p-value ≈ 0.1) were found with higher GPS speed, higher percentage of in-vehicle time, more weekday than weekend measurements, in women who reported work-related exposures, and in women who did not speak English at home.

**Table 1:** **Subject information and subject-level average exposure of PB-PAH (ng/m**^**3**^**) across all the sampling sessions**

		***N (%)***	***Arithmetic mean(STD***^***a***^***)***	***Geometric mean (STD***^***a***^***)***	***P-value***^***b***^	***Range***
Overall		28 (100.0%)	11.0(6.2)	9.3(1.9)		1.7-29.9
Complete days of measurements (≥6 h/day)	1 day	6 (21.4%)	8.1(6.5)	5.7(2.7)	0.17	1.7-15.4
	2-5 days	8 (28.6%)	13.5(7.3)	12.2(1.6)		6.6-29.9
	6-9 days	8 (28.6%)	11.3(6.7)	9.9(1.7)		4.6-24.3
	≥10 days	6 (21.4%)	10.2(3.2)	9.8(1.5)		4.4-13.2
Age	18-29	15 (53.6%)	10.5(6.9)	8.4(2.1)	0.40	1.7-29.9
	30-38	13 (46.4%)	11.5(5.6)	10.4(1.7)		4.4-24.3
Race/Ethnicity	Asian	6 (21.4%)	11.7(4.7)	10.8(1.7)	0.42	4.4-15.8
	Hispanic	13 (46.4%)	10.6(7.3)	8.4(2.2)		1.7-29.9
	Non-Hispanic White	6 (21.4%)	13.4(6.2)	12.4(1.6)		6.6-24.3
	Other	3 (10.7%)	6.2(1.6)	6.2(1.3)		4.6-7.7
Income	Low (<$50 k/year)	15 (53.6%)	10.7(5.6)	9.3 (1.9)	1.00	1.7-24.3
	High (≥$50 k/year)	13 (46.4%)	11.3(7.2)	9.3 (2.0)		2.4-29.9
Speaking English at home	No	11 (39.3%)	12.5(4.9)	11.9(1.4)	0.11	6.6-24.3
	Yes	17 (60.7%)	10.0(7.0)	8.0(2.1)		1.7-29.9
Worker	No	11 (39.3%)	9.2(5.0)	7.6(2.1)	0.19	1.7-15.8
	Yes	17 (60.7%)	12.2(6.8)	10.6(1.8)		2.8-29.9
Had work-related exposure to traffic pollutants^*c*^	No	23(82.1%)	10.1(6.0)	8.5(1.9)	0.10	1.7-29.9
	Yes	5(17.9%)	15.2(6.1)	14.3(1.5)		7.7-24.3
Indoor time	<90.7%	14 (50%)	11.9(7.9)	9.5(2.2)	0.91	1.7-29.9
	≥90.7%	14 (50%)	10.1(4.1)	9.2(1.7)		2.4-16.3
In-vehicle time	<3.8%	14 (50%)	9.3(5.2)	7.6(2.1)	0.11	1.7-16.3
	≥3.8%	14 (50%)	12.6(6.9)	11.3(1.6)		4.6-29.9
Weekday	<72.9%	14 (50%)	9.9(7.5)	7.6(2.2)	0.09	1.7-29.9
	≥72.9%	14 (50%)	12.1(4.7)	11.4(1.4)		6.6-24.3
Daytime	<54.1%	14 (50%)	10.7(6.8)	9.1(1.9)	0.85	2.4-29.9
	≥54.1%	14 (50%)	11.3(5.9)	9.6(2.0)		1.7-24.3
Time in rush hour	<21.6%	14 (50%)	10.8(4.1)	10.1(1.5)	0.54	4.4-16.3
	≥21.6%	14 (50%)	11.2(8.0)	8.6(1.3)		1.7-29.9
GPS speed	<1.9 km/h	14 (50%)	9.1(4.9)	7.6(2.1)	0.10	1.7-16.3
	≥1.9 km/h	14 (50%)	12.8(7.1)	11.4(1.7)		4.6-29.9
Elevation	<26.6 m	14 (50%)	12.0(5.3)	10.6(1.9)	0.30	1.7-24.3
	≥26.6 m	14 (50%)	10.0(7.1)	8.2(2.0)		2.4-29.9
Length-weighted AADT within 500 m	<23172 vehicles/day	14 (50%)	10.2(7.1)	8.1(2.1)	0.28	1.7-29.9
	≥23172 vehicles/day	14 (50%)	11.8(5.4)	10.6(1.7)		2.8-24.3
Traffic density within 300 m	<79891 vehicles per day*km/km^2^	14 (50%)	8.4(4.6)	7.0(2.0)	0.02	1.7-15.4
	≥79891 vehicles per day*km/km^2^	14 (50%)	13.5(6.7)	12.3(1.6)		6.3-29.9
Ambient temperature	<20.6 °C	14 (50%)	9.9(5.9)	8.3(1.9)	0.37	2.4-24.3
	≥20.6 °C	14 (50%)	12.1(6.6)	10.4(1.9)		1.7-29.9
Ambient relative humidity	<63.7%	14 (50%)	13.9(7.0)	12.2(1.8)	0.02	2.8-29.9
	≥63.7%	14 (50%)	8.1(3.9)	7.1(1.9)		1.7-13.2
Wind speed	<4.3 m/s	14 (50%)	11.2(6.6)	9.7(1.8)	0.75	2.4-29.9
	≥4.3 m/s	14 (50%)	10.8(6.2)	8.9(2.1)		1.7-24.3

^*a*^ Standard deviation.

^*b*^*p*-value for the difference in geometric means stratified by different sub-groups.

^*c*^Work-related exposure includes working around parking garage, kiosk, auto shop, buses, trucks, or heavy traffic, or driving a car/bus/truck during the work.

From the minute-level data we found that the subjects spent 91.3% of their time indoors, 4.5% of their time traveling in vehicles, and 4.2% of their time in doing other activities (Additional file 1: Table S2). Due to the large number of observations, the difference in geometric means stratified by different sub-groups were all significant (*p*-value < 0.001) at the minute level. The largest differences among sub-groups were observed by time-activity category (geometric mean 46.8 ng/m^3^ in-vehicle travel vs. 1.9 ng/m^3^ indoor, and 3.2 ng/m^3^ other) and by GPS speed [56.4 ng/m^3^ for speed > 8 km/h (likely in-vehicle travel) vs. 3.0 ng/m^3^ for speed 2–8 km/h (likely outdoor walking and slower in-vehicle travel) vs. 2.0 ng/m^3^ for speed < 2 km/h (likely static indoor or outdoor)] (Additional file 1: Table S1).

### Correlation analysis

Table 2 shows the mean, standard deviation, correlation coefficients of square-root transformed daily PB-PAH concentrations and key predictor variables. The PB-PAH exposure was significantly and positively correlated with weekday, the percentage of data collected during the daytime and while in-vehicle, GPS speed, and traffic-related variables. PB-PAH was significantly but negatively associated with the percentage of data collected during times indoor. The percent of indoor time was strongly and negatively correlated with in-vehicle time, GPS speed, and ambient wind speed, and weakly and negatively correlated with ambient wind speed. The percent of in-vehicle time was strongly and positively correlated with GPS speed and weakly and positively correlated with the percent of daytime. The meteorological variables were not significantly correlated with daily personal PB-PAH exposures.

**Table 2:** Pearson’s correlation coefficients for square root of daily PB-PAH exposures and key predictor variables

	**PB-PAH SQRT**^**a**^	**Indoor time (%)**	**In-vehicle time (%)**	**Weekday (1/0)**	**Daytime (%)**	**Time in rush hour (%)**	**GPS speed (km/h)**	**Elevation (m)**	**Length-weighted AADT within 500 m (vehicles/day)**	Traffic density within 300 m (vehicles per day*km/km^**2**^)	**Ambient temperature (°C)**	**Ambient wind speed (m/s)**	**Ambient humidity (%)**
N	180	180	180	180	180	180	180	180	180	180	180	180	180
Mean	3.0	90.7	4.6	0.73	55.2	22.2	2.3	38.1	35796.5	163362.7	21.1	4.2	63.0
Standard deviation	1.3	7.9	5.1	0.44	18.2	7.0	2.7	80.6	30026.1	211034.8	3.2	1.7	12.4
PB-PAH SQRT^a^	1.00												
Indoor time (%)	−0.55***	1.00											
In-vehicle time (%)	0.69***	−0.81***	1.00										
Weekday (1/0)	0.16*	−0.07	0.04	1.0									
Daytime (%)	0.28***	−0.22**	0.21**	−0.13	1.00								
Time in rush hour (%)	0.07	−0.04	0.03	0.04	0.13	1.00							
GPS speed (km/h)	0.60***	−0.74***	0.93***	−0.06	0.25***	0.01	1.00						
Elevation (m)	−0.08	−0.09	0.07	−0.12	−0.03	−0.06	0.16*	1.00					
Length-weighted AADT within 500 m (vehicles/day)	0.27***	0.06	−0.02	0.08	0.32***	0.03	−0.06	−0.10	1.00				
Traffic density within 300 m (vehicles per day*km/km^2^)	0.19*	0.11	−0.07	0.06	0.31***	0.04	−0.08	−0.09	0.94***	1.00			
Ambient temperature (°C)	0.09	0.06	0.09	−0.02	0.26***	−0.14	0.13	−0.04	0.09	0.07	1.00		
Ambient wind speed (m/s)	0.14	−0.32***	0.17	−0.06	0.53***	0.15*	0.20**	0.07	0.06	0.05	0.07	1.00	
Ambient humidity (%)	−0.11	0.02	−0.09	0.06	−0.37***	0.06	−0.13	0.02	−0.05	−0.01	−0.72***	−0.12	1.00

^*a*^ Square-root transformed PB-PAH concentration.

*p-value <0.05; **p-value < 0.01; ***p-value < 0.001.

### Regression models

Table 3 shows the final selected linear and mixed effect models for estimating daily PB-PAH exposures (square-root transformed). The four-variable linear model explained 59% of the variance in the average daily personal exposures (N = 180 person-days). In particular, percent of in-vehicle time, length-weighted AADT, work-related exposure, and weekday accounted for 48%, 8%, 2%, and 1% of the variances, respectively. The parameter estimates from the linear model and the mixed effect model were almost the same (Table 3). A key advantage of linear model is the ability to estimate an R^2^.

**Table 3:** Regression model for square root of daily average PB-PAH exposures by subject (N = 180)

	**General linear regression**					**Mixed effect model (fixed effects)**		
**Variable**	**Beta**	**Standard error**	***p*-value**	**Partial R**^**2**^	**VIF**	**Beta**	**Standard error**	***p*-value**
Intercept	1.54	0.15	<.0001		0	1.55	0.18	<.0001
Percent of in-vehicle travel time	16.90	1.20	<.0001	0.48	1.01	17.40	1.10	<.0001
Length-weighted AADT within 500 m	1.02*10^-5^	2.10*10^-6^	<.0001	0.08	1.05	0.94*10^-5^	3.07*10^-6^	0.0027
Had work-related exposure to traffic pollutants (yes/no)^*a*^	0.41	0.15	0.0080	0.02	1.05	0.57	0.27	0.0378
Weekday (1/0)	0.33	0.14	0.0192	0.01	1.01	0.31	0.13	0.0173

^*a*^Work-related exposure includes working around parking garage, kiosk, auto shop, buses, trucks, or heavy traffic, or driving a car/bus/truck during the work.

For subject-level PB-PAH exposure (square-root transformed), the best fitting model was a four-variable linear regression model that had an R^2^ of 0.71 and an adjusted R^2^ of 0.66 (Additional file 1: Table S3). Due to the small sample size (N = 28 subjects), we removed the least significant variable (length-weighted AADT with a partial R^2^ of 0.06) from the model and reported a three-variable model in Table 4. The final three-variable model explained 65% of the between-subject variance in PB-PAH exposures. Percentage of in-vehicle travel time, percent of weekday time, and work-related exposure explained 39%, 16%, and 10% of the variance, respectively. When we excluded the subjects with only one day of measurements (N = 6 subjects), the model with the same three variables (somewhat different coefficients) produced a lower R^2^ of 0.50 and an adjusted R^2^ of 0.42 (Additional file 1: Table S4), likely due to a considerable decrease in sample size.

**Table 4:** **Regression model for square root of average PB-PAH exposures of each subject across all the sampling sessions (N = 28; R**^**2**^ **= 0.65; adjusted R**^**2**^ **= 0.61)**

**Variable**	**Beta**	**Standard error**	***p*-value**	**Partial R**^**2**^	**VIF**
Intercept	1.68	0.31	<.0001		0
Percent of in-vehicle travel time	12.19	2.35	<.0001	0.39	1.01
Percent of weekday time	1.14	0.39	0.0078	0.16	1.03
Had work-related exposure to traffic pollutants (yes/no)^*a*^	0.77	0.30	0.0163	0.10	1.03

^*a*^Work-related exposure includes working around parking garage, kiosk, auto shop, buses, trucks, or heavy traffic, or driving a car/bus/truck during the work.

Table 5 shows the linear regression model for estimating subject-level PB-PAH exposures (square-root transformed) in major time-activity categories (N = 74 person-activity categories; adjusted R^2^: 0.75). This was to enhance the potential contrasts in the predictors of PB-PAH rather than to predict PB-PAH in each specific microenvironment since the sample sizes were too small for that. In order to ensure the accurate representativeness of each time-activity category, six records were excluded *a priori* because they lasted for less than 60 min in total across all the sampling sessions for a particular time-activity category. Square-root of GPS speed, indoor (yes/no), and percent of daytime accounted for 67%, 7%, and 2% of the variance in the PB-PAH exposure, respectively. When we included all the records (N = 80 person-activity categories), the model with the same three variables (slightly different coefficients) had a slightly smaller adjusted R^2^ of 0.73 (Additional file 1: Table S5).

**Table 5:** **Regression model for square root of average PB-PAH exposures of each subject in major time-activity categories (indoor, in-vehicle, and other) across all the sampling sessions (N = 74**^***a***^**; R**^**2**^ **= 0.76; adjusted R**^**2**^ **= 0.75)**

	**Beta**	**Standard error**	***p*-value**	**Partial R**^**2**^	**VIF**
Intercept	1.29	1.18	0.2776		0
GPS speed (sqrt)	0.82	0.11	<.0001	0.67	1.62
Indoor (yes/no)	−1.75	0.58	0.0039	0.07	1.72
Percent of daytime	3.87	1.48	0.0108	0.02	1.65

^*a*^We excluded 6 records that lasted for less than 60 min in total for a particular time-activity category (i.e. indoor, in-vehicle travel, and other).

## Discussions

We examined personal PB-PAH exposures by major influential factors and developed regression models to estimate PB-PAH exposures based on GPS-tracking data, traffic activity data, and simple questionnaire information (adjusted R^2^ ranged from 0.58 to 0.75). The strongest predictors of personal PB-PAH exposures were found to be time in-vehicle and the related GPS speed variable, as well as variables describing other exposures to traffic such as traffic density at nearby streets (length-weighted AADT) and work-related exposures to traffic pollutants. The GPS-acquired data made it possible to determine the value of these variables with considerable temporal and spatial accuracy as we reported previously [23]. Our study adds important new findings to the literature on PB-PAH exposure assessment in several ways: (1) it is one of the first studies to model personal PB-PAH exposures at a high-temporal resolution; (2) it demonstrated the usefulness of coupling real-time exposure measures with GPS tracking in personal air pollution exposure assessment; and 3) it confirmed the importance of GPS-based time-activity and GPS speed as a surrogate of on-road exposures in PB-PAH exposure modeling.

Our personal PB-PAH measurements revealed an undeniable contribution from the transport microenvironment. The amount of time in vehicle (on average 4.5% of the total sampling time) explained 48% of the variance in daily personal PB-PAH exposure and 39% of the variance in subject-level exposure. Time in vehicle was the most important determinant of personal PB-PAH exposure, which confirms earlier studies on the relationship between activities and traffic-related air pollution exposure that have suggested an important role for the traffic microenvironment, despite the limited time spent in or near traffic environments [29,46,47]. Significant exposure misclassification may occur if only residential exposures are considered since time spent in or near transport may provoke dissimilarity in personal exposure between individuals with similar residential exposures. In addition to in-vehicle time, length-weighted AADT within 500 m explained 8% of the variance in daily PB-PAH exposure. This variable explained approximately 28% of the variance in subject-level indoor PB-PAH exposures (data not shown), which confirms the importance of local traffic emissions to the exposures not only in the commuting environment but also indoors. Finally, the in-vehicle time and GPS speed variables were highly correlated (r > 0.90 for daily and subject-level data). In-vehicle time tended to correlate slightly better with daily and subject-level total exposures, although the square root of GPS speed was a better predictor in the subject-level microenvironmental model (Table 5) and explained 67% of the variance.

The work-related exposure to traffic-related pollutants explained 10% of the variance in PB-PAH exposure at the subject level and 2% of the variance in daily exposure. The work-related exposures were obtained from the questionnaire based on the typical activity patterns in the past three months of the personal sampling, thus this variable was not able to capture substantial day-to-day variation in subjects’ time activity patterns and PB-PAH exposures. Furthermore, approximately 40% of women did not work, thus their activity patterns may vary considerably on a daily basis compared to full-time workers. We also found that subjects tended to have higher PB-PAH exposures during weekdays than weekends and thus the percent of weekday time explained 16% of the variance in PB-PAH exposures at the subject level. Previous studies have reported higher ambient concentrations of traffic-related pollutants on weekdays [48,49], but little is known about the day of week impact on personal exposures. In addition, we found women who did not speak English at home had marginally higher PB-PAH exposures although this variable was not selected in the final prediction models. Further investigation of our data showed that women who did not speak English at home had a higher percent of in-vehicle time than the others (5.9% vs. 4.1%) although the difference was not significant (*p*-value = 0.35).

We found that the percent of indoor time was negatively associated with PB-PAH exposure, which is expected due to few PB-PAH sources indoors in this study. Although we did observe and remove high PB-PAH concentrations indoors (e.g. five 1-min exposures exceeding 1000 ng/m^3^), such events had little influence on the overall average exposures since they occurred occasionally and lasted for a very short period (e.g. one minute). In fact, the models changed little when we included the five outliers with extremely high indoor concentrations (data not shown). This lack of impact on PB-PAH by the indoor environment is likely because we only recruited non-smokers and the prevalence of other indoor sources (e.g. ETS, wood-burning, grilling, and barbecue) was relatively low. California had the 2^nd^ lowest smoking rate (14% in adult population) among all the states in the U.S. [50] and smoking was prohibited in almost all indoor and outdoor public places in California [51]. Indoor wood-burning or indoor grill/barbecue was not common because of the warm climate of southern California and that our samples were mostly collected in the warm season. However, in other regions or other populations, these non-traffic related indoor sources may contribute more to personal PB-PAH exposures than what was shown in our study.

The geometric mean of PB-PAH exposures tended to be higher in rush hours than non-rush hours at the minute level (Additional file 1: Table S1), but the pattern was opposite at the subject level (not statistically significant) (Table 1). We found that the geometric mean of one-minute PB-PAH concentrations started to increase remarkably at 6 AM, peaked at 7 AM (geometric mean = 5.1 ng/m^3^), dropped gradually from 9 AM to 2 PM, peaked again at 4 PM (geometric mean = 3.4 ng/m^3^), and started to drop gradually from 5 PM till 1 AM in the morning (Additional file 1: Table S6). We observed a late afternoon peak (3–5 PM) in PB-PAH exposure, but not the evening rush hour peak that occurs at 4–7 PM. Since about 40% of the study participants did not work, they may have picked up their children or done errands in the late afternoon or other times of the day. We found that on average the subjects spent approximately 9.8% and 7.1% of the time traveling in vehicles during 3–5 PM and 4–7 PM, respectively. Thus, the rush hour variable may not appropriately capture their time in traffic.

No meteorological variables were entered into the predictive models. This is likely because we modeled personal exposure rather than ambient outdoor pollutant concentrations. Personal exposures are strongly influenced by near-source activities of human subjects. Although subject-level PB-PAH exposure was significantly higher (r = 0.02) in the group with lower relative humidity (Table 1), the continuous measure of relative humidity was only marginally correlated with subject-level exposure (r = 0.10) and was not selected in the final models. In addition to wind, temperature, and relative humidity, we also examined the usefulness of the atmospheric stability class data modeled every three hours at 40 km by 40 km resolution from the nearest EDAS modeling grid of the National Oceanic and Atmospheric Administration (http://www.arl.noaa.gov/ready.html). However, the stability variables were not significantly associated with PB-PAH exposures, likely due to substantial uncertainties associated with the modeled stability estimate.

A major limitation of the study is the semi-quantitative feature of the PAS sampler. The PAS may respond differently to individual PAH species thus the PAS signal may not be directly proportional to the concentration of individual species [34]. In addition, the components of the PAH mixtures may differ by emission sources (e.g. traffic, tobacco smoke, wood combustion, food grill), thus the PAS measurements reflect not only the total concentration but also the nature of the PAH mixtures in different microenvironments. This creates uncertainty in the exposure measures among different microenvironments. Despite the limitations, the PAS sampler is the only available instrument that is capable of continuous personal PB-PAH monitoring. The highly informative nature of the predictive models (adjusted R^2^: 0.58-0.75) is a testament to the approach, which could be adapted to methods using more accurate instruments in the future if they become available.

Other time-activity patterns such as biking and travel by bus and subways may also be associated with high levels of exposure to traffic-related air pollutants including PB-PAH [52-54]. However, no subjects reported traveling in an underground train, by bus, or biking based on our questionnaire on the means of transportation. Therefore, we did not examine the other travel modes in this study although they may be important in other studies and regions where subjects may engage in these activities frequently.

We did not use diaries to track subjects’ activities that may significantly influence their exposure levels (e.g. near a smoker, cooking) but are not easily obtained from the GPS data alone, mainly because the collection of such detailed information may significantly increase the burden to the subjects. Combining the GPS data with simple questionnaire or diary data may further improve the model performance. Additionally, our subjects were only pregnant women or women who had delivered babies within one year of the sampling dates. Other population groups (e.g. children, men, other women) and subjects in other regions may have different time-activity patterns than our study participants. However, we believe that the method of coupling real-time exposure sampling with GPS time-activity tracking and the application of GPS data in exposure modeling can be easily adapted to other populations in different studies.

We found higher PB-PAH exposures in the winter with a geometric mean (based on one-minute data) of 4.8 ng/m^3^ and 2.3 ng/m^3^ in the cool and warm season, respectively (Additional file 1: Table S2). Unfortunately, less than 5% of our data were collected during the cool season. Thus we could not examine the seasonal difference. Future research may improve model prediction by sampling in different seasons and measuring a more diverse and larger number of subjects.

## Conclusions

We developed regression models to estimate PB-PAH exposures based on GPS-tracking data, traffic activity and roadway data, and simple questionnaire information (adjusted R^2^ ranging from 0.58 to 0.75). Time in vehicle was the most important determinant of personal PB-PAH and explained 48% of the variance in daily personal PB-PAH exposure and 39% of the variance in subject-level exposure. The other major predictors included length-weighted traffic count, work-related exposure to traffic-related pollutants, and percent of weekday time. We demonstrated the importance of coupling real-time exposure measures with GPS time-activity tracking in personal air pollution exposure assessment. The methods presented here can be applied to large epidemiologic cohort studies interested in the effects of PB-PAH exposure where there is also a desire to limit participant burden.

## Abbreviations

GPS, Global positioning systems; PB-PAH, Particle-bound polycyclic aromatic hydrocarbon; PAS, Photoelectric aerosol sensor.

## Competing interests

The authors declare that they have no competing interests.

## Authors' contributions

JW is the PI of this study. JW conceptualized the study, developed the methods, analyzed the data, and drafted the manuscript. TT and LL helped in data analysis. GJ played a major role in collecting the PB-PAH and GPS measurement data. RD helped conceptualize the study and provided important intellectual content for the manuscript. All authors assisted in the interpretation of results and contributed towards the final version of the manuscript. All authors read and approved the final manuscript.

## Supplementary Material

Additional file 1 **Table S1.** Distribution of PAH exposure. Table S2. Summary of one-minute PAH exposure levels (ng/m^3^) by key variables. Table S3. Regression model for square root of average PAH exposures of each subject across all the sampling sessions – four-variable model (N = 28; R^2^ = 0.71; adjusted R^2^ = 0.66). Table S4. Regression model for square root of average PAH exposures of each subject across all the sampling sessions (six subjects with only one-day of measurement data excluded. N = 22; R^2^ = 0.50; adjusted R^2^ = 0.42). Table S5. Regression model for square root of average PAH exposures of each subject in major time-activity categories (indoor, in-vehicle, and other) across all the sampling sessions (N = 80; R^2^ = 0.74; adjusted R^2^ = 0.73). Table S6. Diurnal distribution of geometric means of one-minute PAH exposures (ng/m^3^). Figure S1. The non-linear relationship between square root of daily PAH exposure and average GPS speed. Click here for file
