# Supplementary material for: Modeling personal particle-bound polycyclic aromatic hydrocarbon (pb-pah) exposure in human subjects in Southern California
Source: Environ Health. 2012 Jul 11;11:47. doi: 10.1186/1476-069X-11-47 (PMC3436775; doi:10.1186/1476-069X-11-47)
Supplement: Additional file 1 — Table S1. Distribution of PAH exposure. Table S2. Summary of one-minute PAH exposure levels (ng/m3) by key variables. Table S3. Regression model for square root of average PAH exposures of each subject across all the sampling sessions – four-variable model (N = 28; R2 = 0.71; adjusted R2 = 0.66). Table S4. Regression model for square root of average PAH exposures of each subject across all the sampling sessions (six subjects with only one-day of measurement data excluded. N = 22; R2 = 0.50; adjusted R2 = 0.42). Table S5. Regression model for square root of average PAH exposures of each subject in major time-activity categories (indoor, in-vehicle, and other) across all the sampling sessions (N = 80; R2 = 0.74; adjusted R2 = 0.73). Table S6. Diurnal distribution of geometric means of one-minute PAH exposures (ng/m3). Figure S1. The non-linear relationship between square root of daily PAH exposure and average GPS speed. [file 1476-069X-11-47-S1.doc]

Table S1. Distribution of PAH exposures

|  | Skewness | | | Kolmogorov-Smirnov | | |
| --- | --- | --- | --- | --- | --- | --- |
|  | PAH | PAH_sqrt | PAH_log | PAH | PAH_sqrt | PAH_log |
| Minute-level exposure (N=185,662) | 8.76 | 4.30 | 1.07 | <0.01 | <0.01 | <0.01 |
| Daily exposure (N=180) | 1.74 | 0.58 | -0.27 | <0.01 | 0.08 | 0.03 |
| Subject-level exposure (N=28) | 1.10 | 0.10 | -0.87 | >0.15 | >0.15 | >0.15 |

Table S2. Summary of one-minute PAH exposure levels (ng/m^3^) by key variables

|  |  | N (%) | Arithmetic Mean(STD) | Geometric Mean*^a^* (STD) | Median | Range |
| --- | --- | --- | --- | --- | --- | --- |
| Overall | 185662 (100.0%) | | 10.6(41.4) | 2.3(4.8) | 2.3 | 0-1078 |
| Complete days of measurements (≥6 hours/day) | 1 day | 3646 (2.0%) | 7.9(37.0) | 1.3(5.8) | 2.0 | 0-717 |
|  | 2-5 days | 27594 (14.9%) | 12.0(44.2) | 3.4(4.0) | 3.3 | 0-947 |
|  | 6-9 days | 69024 (37.2%) | 10.5(42.9) | 1.9(5.1) | 2.0 | 0-1078 |
|  | ≥10 days | 85398 (46.0%) | 10.4(39.3) | 2.4(4.6) | 2.3 | 0-951 |
| Age | 18-29 | 78444 (42.3%) | 10.9(41.8) | 2.8(4.4) | 2.6 | 0-1078 |
|  | 30-38 | 107218 (57.7%) | 10.4(41.1) | 2.0(5.0) | 2.0 | 0-952.6 |
| Race/Ethnicity | Asian | 38755 (20.9%) | 9.4(39.6) | 1.9(5.0) | 2.0 | 0-1078 |
|  | Hispanic | 70202 (37.8%) | 11.4(41.3) | 2.7(4.6) | 2.6 | 0-889.3 |
|  | Non-Hispanic White | 51172 (27.6%) | 12.6(48.4) | 2.6(4.6) | 2.6 | 0-952.6 |
|  | Other | 25533 (13.8%) | 6.3(25.7) | 1.5(4.7) | 1.6 | 0-666.6 |
| Income | Low (<$50 k/year) | 92961 (50.1%) | 9.7(37.8) | 2.2(4.7) | 2.3 | 0-952.6 |
|  | High (≥$50 k/year) | 92701 (49.9%) | 11.5(44.7) | 2.3(4.9) | 2.3 | 0-1245 |
| Speaking English at home | No | 72569 (39.1%) | 11.8(43.8) | 2.4(4.9) | 2.3 | 0-952.6 |
|  | Yes | 113093 (60.9%) | 9.8(39.7) | 2.2(4.7) | 2.3 | 0-1078 |
| Worker | No | 51176 (27.6%) | 9.4(38.5) | 2.4(4.6) | 2.3 | 0-951 |
|  | Yes | 134486 (72.4%) | 11.0(42.4) | 2.3(4.8) | 2.3 | 0-1078 |
| Had work-related exposure to traffic pollutants*^b^* | No | 144613 (77.9%) | 9.7(39.5) | 2.2(4.7) | 2.3 | 0-1078 |
|  | Yes | 41049 (22.1%) | 13.7(47.2) | 2.8(5.1) | 2.6 | 0-952.6 |
| Pregnancy status | No | 72575 (39.1%) | 11.4(41.7) | 2.5(4.8) | 2.3 | 0-947 |
|  | Yes | 113087 (60.9%) | 10.1(41.2) | 2.2(4.7) | 2.3 | 0-1078 |
| GPS-based time activity category | Indoor | 169589 (91.3%) | 5.4(24.3) | 1.9(4.0) | 2.0 | 0-952.6 |
|  | In-vehicle travel | 8326 (4.5%) | 103.7(108.9) | 46.8(5.3) | 71.3 | 0-1078 |
|  | Other | 7747 (4.2%) | 23.3(72.7) | 3.2(6.4) | 2.6 | 0-860.3 |
| Season | Warm (May-October) | 177066 (95.4%) | 10.2(40.9) | 2.2(4.7) | 2.3 | 0-1078 |
|  | Cool (November-April) | 8596 (4.6%) | 18.4(49.6) | 4.6(5.6) | 4.8 | 0-742.3 |
| Day of week | Weekend | 56172 (30.3%) | 8.7(35.8) | 1.9(4.6) | 2.0 | 0-840.6 |
|  | Weekday | 129490 (69.7%) | 11.4(43.6) | 2.5(4.8) | 2.3 | 0-1078 |
| Daytime | 0 (7 PM-6 AM) | 82789 (44.6%) | 4.4(20.3) | 1.7(3.8) | 2.0 | 0-890.3 |
|  | 1 (6 AM-7PM) | 102873 (55.4%) | 15.6(52.0) | 2.9(5.4) | 2.6 | 0-1078 |
| Time in rush hour | 0 (Other times) | 145549 (78.4%) | 8.5(36.3) | 2.1(4.4) | 2.3 | 0-1078 |
|  | 1 (6-8 AM and 4-7 PM) | 40113 (21.6%) | 18.1(55.4) | 3.1(5.9) | 2.6 | 0-952.6 |
| GPS speed | <2 km/h | 171191 (92.2%) | 6.0(27.0) | 2.0(4.0) | 2.3 | 0-1078 |
|  | 2-8 km/h | 7351 (4.0%) | 23.2(73.3) | 3.0(6.3) | 2.6 | 0-951 |
|  | >8 km/h | 7120 (3.8%) | 108.7(106.2) | 56.4(4.5) | 78.0 | 0-947 |
| Elevation(m) | <50th percentile | 92830 (50.0%) | 12.4(46.4) | 2.5(5.0) | 2.3 | 0-1078 |
|  | 50^th^-75^th^ percentile | 46417 (25.0%) | 10.0(38.5) | 2.7(4.3) | 2.6 | 0-951 |
|  | >75th percentile | 46415 (25.0%) | 7.5(32.4) | 1.7(4.6) | 2.0 | 0-894 |
| Length-weighted AADT within 500 m | <50th percentile | 92827 (50.0%) | 8.6(37.2) | 2.0(4.6) | 2.0 | 0-1078 |
|  | 50^th^-75^th^ percentile | 46415 (25.0%) | 7.7(35.0) | 1.7(4.5) | 2.0 | 0-894 |
|  | 75-90th percentile | 27852 (15.0%) | 15.6(51.8) | 3.4(4.6) | 3.0 | 0-951 |
|  | >90th percentile | 18568 (10.0%) | 20.5(54.5) | 5.4(4.8) | 4.6 | 0-952.6 |
| Traffic density within 300 m | <50th percentile | 92943 (50.1%) | 6.7(32.2) | 1.8(4.2) | 2.0 | 0-1078 |
|  | 50^th^-75^th^ percentile | 46359 (25.0%) | 8.4(36.3) | 1.8(4.7) | 2.0 | 0-952.6 |
|  | 75-90th percentile | 27817 (15.0%) | 22.7(64.3) | 4.7(5.2) | 4.0 | 0-951 |
|  | >90th percentile | 18543 (10.0%) | 17.2(46.1) | 4.6(4.7) | 4.0 | 0-832.6 |
| Ambient temperature | <18.3 ºC | 42805 (23.1%) | 6.6(25.1) | 2.2(4.1) | 2.3 | 0-742.3 |
|  | 18.3-20.6 ºC | 49070 (26.4%) | 9.2(40.3) | 1.9(4.6) | 2.0 | 0-952.6 |
|  | 20.6-23.3 ºC | 43675 (23.5%) | 11.1(43.0) | 2.2(4.8) | 2.3 | 0-947 |
|  | >23.3 ºC | 50112 (27.0%) | 15.0(50.7) | 2.9(5.3) | 2.6 | 0-1078 |
| Ambient relative humidity | <18.3 % | 44439 (23.9%) | 14.3(48.1) | 3.0(5.2) | 2.6 | 0-1078 |
|  | 18.3-20.6 % | 43060 (23.2%) | 12.9(46.8) | 2.5(5.1) | 2.3 | 0-947 |
|  | 20.6-23.3 % | 44499 (24.0%) | 10.0(40.6) | 2.1(4.8) | 2.0 | 0-952.6 |
|  | >23.3 % | 53664 (28.9%) | 6.1(29.2) | 1.8(4.0) | 2.0 | 0-890.3 |
| Ambient wind speed | 0 m/s | 63981 (34.5%) | 7.8(29.4) | 2.3(4.3) | 2.3 | 0-947 |
|  | 0.1-5 m/s | 28478 (15.3%) | 8.3(33.4) | 2.0(4.5) | 2.0 | 0-719 |
|  | 5-7 m/s | 41605 (22.4%) | 11.1(44.0) | 2.2(4.9) | 2.3 | 0-951 |
|  | >7 m/s | 51598 (27.8%) | 14.9(53.7) | 2.5(5.3) | 2.3 | 0-1078 |

*^a^* All the p-value were <0.001 for the difference in geometric means stratified by different sub-groups.

*^b^* Work-related exposure includes working around parking garage, kiosk, auto shop, buses, trucks, or heavy traffic, or driving a car/bus/truck during the work.

Table S3. Regression model for square root of average PAH exposures of each subject across all the sampling sessions – four-variable model (N=28; R^2^=0.71; adjusted R^2^=0.66)

| Variable | Beta | Standard error | p-value | Partial R^2^ | VIF |
| --- | --- | --- | --- | --- | --- |
| Intercept | 1.48 | 0.30 | <.0001 |  | 0 |
| Percent of in-vehicle travel time | 12.51 | 2.19 | <.0001 | 0.39 | 1.01 |
| Percent of weekday time | 1.02 | 0.37 | 0.0110 | 0.16 | 1.05 |
| Had work-related exposure to traffic pollutants*^a^* | 0.65 | 0.28 | 0.0293 | 0.10 | 1.06 |
| Length-weighted AADT in 500 m | 8.45*10^-6^ | 3.82*10^-6^ | 0.0371 | 0.06 | 1.07 |

*^a^* Work-related exposure includes working around parking garage, kiosk, auto shop, buses, trucks, or heavy traffic, or driving a car/bus/truck during the work.

Table S4. Regression model for square root of average PAH exposures of each subject across all the sampling sessions (six subjects with only one-day of measurement data excluded. N=22; R^2^=0.50; adjusted R^2^=0.42)

| Variable | Beta | Standard error | p-value | Partial R^2^ | VIF |
| --- | --- | --- | --- | --- | --- |
| Intercept | 1.55 | 0.98 | 0.1326 |  | 0 |
| Percent of in-vehicle travel time | 14.37 | 4.07 | 0.0024 | 0.38 | 1.85 |
| Percent of weekday time | 1.31 | 1.12 | 0.2594 | 0.08 | 1.85 |
| Had work-related exposure to traffic pollutants*^a^* | 0.59 | 0.35 | 0.1077 | 0.04 | 1.00 |

*^a^* Work-related exposure includes working around parking garage, kiosk, auto shop, buses, trucks, or heavy traffic, or driving a car/bus/truck during the work.

Table S5. Regression model for square root of average PAH exposures of each subject in major time-activity categories (indoor, in-vehicle, and other) across all the sampling sessions (N=80; R^2^=0.74; adjusted R^2^=0.73)

| Variable | Beta | Standard error | p-value | Partial R^2^ | VIF |
| --- | --- | --- | --- | --- | --- |
| Intercept | 0.61 | 1.20 | 0.6151 |  | 0 |
| Speed_sqrt | 0.81 | 0.11 | <.0001 | 0.62 | 1.51 |
| Indoor (yes/no) | -1.71 | 0.61 | 0.0060 | 0.03 | 1.67 |
| Percent of daytime | 4.98 | 1.49 | 0.0013 | 0.09 | 1.61 |

Table S6. Diurnal distribution of geometric means of one-minute PAH exposures (ng/m^3^)

| Hour | N | Arithmetic  Mean(STD) | Geometric  Mean^a^ (STD) | Median | Range |
| --- | --- | --- | --- | --- | --- |
| 0 | 7273 | 2.6(2.6) | 1.6(3.4) | 2.0 | 0-24.6 |
| 1 | 7198 | 2.6(2.7) | 1.5(3.6) | 2.0 | 0-19 |
| 2 | 7212 | 2.6(2.8) | 1.5(3.6) | 1.6 | 0-22.6 |
| 3 | 7238 | 2.6(2.9) | 1.5(3.6) | 1.6 | 0-20.6 |
| 4 | 7100 | 3.7(11) | 1.7(3.8) | 2.0 | 0-296 |
| 5 | 7103 | 6.1(20.7) | 2.2(4.2) | 2.3 | 0-473.3 |
| 6 | 7079 | 20.7(52.6) | 4.4(5.9) | 3.6 | 0-630.6 |
| 7 | 7054 | 22.1(57.1) | 5.1(5.3) | 4.3 | 0-947 |
| 8 | 7309 | 12.2(38.6) | 3.8(4.4) | 3.6 | 0-807 |
| 9 | 7400 | 13.8(48.1) | 3.6(4.4) | 3.6 | 0-889.3 |
| 10 | 7691 | 11.5(37.8) | 3.2(4.2) | 3.0 | 0-647.6 |
| 11 | 7682 | 15.6(55.1) | 3(4.9) | 3.0 | 0-838.3 |
| 12 | 7830 | 15.2(50.3) | 2.7(5.3) | 2.6 | 0-1078.3 |
| 13 | 7910 | 13.2(49.2) | 2.3(5.2) | 2.3 | 0-765.6 |
| 14 | 8363 | 13(52.7) | 2.2(5.2) | 2.0 | 0-834.3 |
| 15 | 8575 | 17.3(58.7) | 2.5(5.9) | 2.3 | 0-894 |
| 16 | 8769 | 26(72.6) | 3.4(7) | 2.6 | 0-952.6 |
| 17 | 8680 | 12.7(46.9) | 2.2(5.2) | 2.0 | 0-799.6 |
| 18 | 8531 | 10(40.4) | 1.9(5) | 2.0 | 0-951 |
| 19 | 8272 | 8(45.5) | 1.8(4.3) | 2.0 | 0-890.3 |
| 20 | 8260 | 4.9(21.5) | 1.7(3.8) | 2.0 | 0-642.6 |
| 21 | 7979 | 5.8(22.5) | 1.8(4.1) | 2.0 | 0-490.5 |
| 22 | 7740 | 5.2(24) | 1.7(3.8) | 2.0 | 0-628 |
| 23 | 7414 | 3(7.8) | 1.6(3.5) | 2.0 | 0-343 |

1. (b)

Figure S1. The non-linear relationship between square root of daily PAH exposure and average GPS speed.
